# Temporal Information Processing in Short- and Long-Term Memory of Patients with Schizophrenia

**DOI:** 10.1371/journal.pone.0026140

**Published:** 2011-10-28

**Authors:** Steffen Landgraf, Joerg Steingen, Yvonne Eppert, Ulrich Niedermeyer, Elke van der Meer, Frank Krueger

**Affiliations:** 1 Department of Psychology, Humboldt-Universität zu Berlin, Berlin, Germany; 2 Inserm-Laboratory of Psychopathology and Mental Diseases, Center for Psychiatry and Neuroscience, U984, Sainte Anne Hospital, Service-Hospitalo Universitaire, Paris, France; 3 Department of Psychiatry and Psychotherapy, Frankfurt, Germany; 4 Department of Molecular Neuroscience, Krasnow Institute for Advanced Study, George Mason University, Fairfax, Virginia, United States of America; 5 Department of Psychology, George Mason University, Fairfax, Virginia, United States of America; French National Centre for Scientific Research, France

## Abstract

Cognitive deficits of patients with schizophrenia have been largely recognized as core symptoms of the disorder. One neglected factor that contributes to these deficits is the comprehension of time. In the present study, we assessed temporal information processing and manipulation from short- and long-term memory in 34 patients with chronic schizophrenia and 34 matched healthy controls. On the short-term memory temporal-order reconstruction task, an incidental or intentional learning strategy was deployed. Patients showed worse overall performance than healthy controls. The intentional learning strategy led to dissociable performance improvement in both groups. Whereas healthy controls improved on a performance measure (serial organization), patients improved on an error measure (inappropriate semantic clustering) when using the intentional instead of the incidental learning strategy. On the long-term memory script-generation task, routine and non-routine events of everyday activities (e.g., buying groceries) had to be generated in either chronological or inverted temporal order. Patients were slower than controls at generating events in the chronological routine condition only. They also committed more sequencing and boundary errors in the inverted conditions. The number of irrelevant events was higher in patients in the chronological, non-routine condition. These results suggest that patients with schizophrenia imprecisely access temporal information from short- and long-term memory. In short-term memory, processing of temporal information led to a reduction in errors rather than, as was the case in healthy controls, to an improvement in temporal-order recall. When accessing temporal information from long-term memory, patients were slower and committed more sequencing, boundary, and intrusion errors. Together, these results suggest that time information can be accessed and processed only imprecisely by patients who provide evidence for impaired time comprehension. This could contribute to symptomatic cognitive deficits and strategic inefficiency in schizophrenia.

## Introduction

Cognitive deficits are to be recognized as core disturbances in patients with schizophrenia [1]–[3]. A large variety of impaired cognitive domains has been identified in patients, including but not limited to executive [4], [5], attention [6]–[9], and memory functions [10]–[13]. However, patients' deficits become most apparent when task demands do not coincide with patients' cognitive strategies [14], [15], implying that deficits might actually be due to an ineffective use of available information. The present study tested how patients with schizophrenia strategically use available temporal information by assessing short-term and long-term memory retrieval.

Seeman [16] explicitly associated schizophrenia patients' reality distortions with cognitive dysfunctions that were due to the dysfunctional concept of time. According to Weinberger et al. [17], hypofrontality in schizophrenia is due to a decreased recruitment of the PFC and a disturbed communication between the PFC and the hippocampus. Previous neuroimaging [18]–[24] and neuropsychological [25]–[36] studies have demonstrated that the prefrontal cortex is involved in the management of script-event knowledge, that is, the retrieval and sequencing of temporal information. Further, the hippocampus is involved in processing information of new temporal sequences, which enables the motor-perceptual system to simulate the actions of others [37], [38]. A large number of studies have shown functional [39]–[43] and structural [44]–[47] abnormalities in the prefrontal cortex, as well as in the hippocampus [48]–[50] in patients with schizophrenia. Therefore, a prefrontal-hippocampal disturbance fits with the assumption of impaired temporal information processing in schizophrenia.

Short-term memory for temporal information is impaired in patients with schizophrenia. Using a temporal-order reconstruction task, Elvevag et al. [13] presented participants two lists of 15 words each. After each list, participants had to reconstruct the temporal order from an array of randomly ordered words. Patients showed poorer short-term memory for the temporal order of words compared to healthy controls. However, patients were also impaired in the recognition and recall of the actual words. The authors reported that covarying recall, but not recognition, eliminated temporal-order reconstruction deficits in patients. Hence, they concluded that a deficit in short-term memory for temporal information and for general word recall might be due to a third process underlying these aberrations. This process, as we and other researchers have argued elsewhere, could resemble patients' inefficient implementation of cognitive and encoding strategies [14], [15], [51]–[53] and, thus, of available temporal information. To test this assumption, we administered a temporal-order reconstruction task that allowed us implement incidental and intentional temporal information encoding strategies.

Encoding and retrieval of temporal information from long-term memory is also impaired in patients with schizophrenia. Temporal information from episodic and long-term memory [54]–[57] enables us to efficiently select adequate behavioral options for social and environmental contexts [58]. Multiple studies have demonstrated deficits in the action representation of patients [59]–[62] in planning action and event sequences [63]–[65], in action monitoring, e.g., [61], and, at an even more basic level, in motion detection [59], [66], [67]. In studies on action representation, Zalla et al. [35] presented videos of action sequences to patients with schizophrenia. Participants had to segment these sequences into meaningful units. Patients had problems dissociating large action segments. This impairment was, further, correlated with disorganisation symptoms and thought disorder. In another study by the same research group [32], patients showed difficulties in generating and organizing long-term memories of so-called scripts. Scripts are mental representations of everyday activities, for example, grocery shopping or going out to a restaurant. These scripts consist of single events usually occurring in a typical temporal order and carrying decisive information about actors, actions, goal hierarchies, and temporal successions [68]. In the chronological action-generation and action-ordering task by Zalla et al. [32], patients showed slower action generation speed, erroneous action sequencing, and impaired action prioritizing with reference to the overall action goal. Because patients showed impairments in planning, problem solving, and goal-directed behavior, e.g., [69], it could be hypothesized that retrieval of temporal information from script knowledge should be specifically impaired in patients if temporal information had to be retrieved in the less favored inverse compared to the chronological temporal direction [27], [28], [32]. Patients might not be able to adapt their problem-solving strategies to these task demands, resulting in behavioral deficits.

The main goal of the present study was to investigate whether patients' deficits in temporal-order reconstruction and script generation could be ascribed to impaired access and processing of temporal information. We administered an episodic short-term and a long-term memory task: the temporal-order reconstruction task adapted from Mangels [70] and the script-generation task adapted from Rosen et al. [71]. In the temporal-order reconstruction task, two lists of twenty words (four words from five semantic categories each) were presented. There were no instructions for the first list, eliciting an “incidental learning strategy.” For the second list, participants were instructed to memorize the order of words, which resulted in an “intentional learning strategy.” Incidental learning was assumed to spontaneously elicit the encoding of items according to semantic content. In the intentional learning condition, participants were expected to use temporal-order information of the presented word list in addition to the semantic information. We hypothesized that temporal-order recollection would be most impaired in patients in the intentional encoding condition due to their impaired implementation of episodic temporal information in this condition.

Using the script-generation task, long-term temporal memory of over-learned action sequences was assessed. Participants had to verbally generate as many actions as possible for two routine and two non-routine scripts either in the chronological or inverse order. We hypothesized that the generation of the scripts and the number of errors would depend on the availability of temporal-order information. Patients were expected to be slower at generating events. Further, we expected, on the one hand, more sequencing and script boundary errors in patients compared to healthy controls, specifically in the inverse condition because temporal information had to be manipulated in this condition. Moreover, patients were expected to show a higher number of irrelevant intrusion errors (events that do not belong to the script) than healthy controls. This was expected to be the case especially in the non-routine conditions, as these conditions provide more opportunities to digress from the normal script due to inflexible focusing on task-irrelevant attention-capturing details [15]. In fact, we expected the number of intrusions to correlate with symptomatology in patients.

## Materials and Methods

### Participants

Thirty-four out-patients (11 female) fulfilling paranoid schizophrenia diagnostic criteria (F20.0) of the ICD-10 [72] were recruited from five different psychiatric hospitals in the federal state of Brandenburg, Germany. Thirty-four healthy controls were selected individually, corresponding to patients' demographic characteristics regarding age, gender, intelligence, and handedness. A multiple-choice vocabulary test (“Mehrfach-Wortschatz-Test”; [73] was used to estimate the intelligence quotient (IQ). Handedness was assessed with the Edinburgh Inventory [74]. Table 1 summarizes demographic and clinical data for both groups. The number of males and females as well as the number of dextral and sinistral individuals was identical in the two groups. Further, groups did not differ in age (t(66) = −0.12, P>0.05) and IQ (t(66) = −0.28, P>0.05). All patients had been on stable atypical antipsychotic medication (olanzapine 5–20 mg/day or risperidone 2–6 mg/day or amisulpride 100–800 mg/day) six months prior to the day of testing. Symptomatology of patients was assessed using the Positive and Negative Syndrome Scale (PANSS; [75] on the day of testing. Exclusion criteria for patients were ophthalmologic, neurological, or cardiovascular diseases, substance abuse or dependence, extrapyramidal symptoms, head trauma, or birth complications. Exclusion criteria for healthy control individuals were a personal history of psychiatric disorders or a family history of psychiatric disorders up to second degree relatives. Prior to participating, each individual provided written informed consent. Participants were debriefed after the experiment. The study was approved by the local Institutional Review Boards.

**Table 1:** Demographic information of the two groups.

	SZ	C
N	34	34
Gender	11F	11F
Age (years)	31.1 (10.8)	31.4 (11.4)
Handedness	31R/3L	31R/3L
IQ	105.2 (11)	104.3 (12)
Disease onset	24.5 (6.9)	-
Disease duration	6.7 (6.9)	-
PANSS positive	21.4 (8.8)	-
PANSS negative	25.9 (10.1)	-
PANSS general	46.4 (13.6)	-
CPZ	181 (98)	-

Abbreviations: N = number of subjects; SZ = chronic schizophrenia patients; C = healthy controls; F = females; R = right-handed; L = left-handed; M = mixed-handed; years of study = 12 years of study corresponding to a high school diploma (Bacheleaureat); PANSS = Positive and Negative Syndrome Scale [75]; CPZ = Chlorpromazine equivalent of daily medication intake; numbers in brackets = standard deviations.

### Methods

Participants were each tested individually in one experimental session that comprised two tasks: The temporal-order reconstruction task, always administered first, followed by the script-generation task. Completion of both tasks took about 50 minutes.

#### Temporal-order reconstruction task

Stimuli. The temporal-order reconstruction task was adapted from Mangels [70]. Stimuli consisted of two lists of 20 common, one- to three-syllable German nouns: the incidental and the intentional learning lists (Table 2). Word frequency obtained from CELEX [76] was comparable between lists (incidental list mean: 5.4 per million (SE = 2.2); intentional list mean: 6.9 per million (SE = 1.7); t(38) = −0.548, P>0.05). Each list was composed of five items from four different semantic categories (incidental: furniture, instruments, kitchen utensils, and weapons; intentional: landscape, clothes, animals, and vegetables). In each list, there were three instances in which two items from the same category were presented sequentially (underlined items in Table 2). Note that these three instances did not occur in immediate succession of each other or within the first or last four positions of the list.

**Table 2:** The incidental and intentional learning lists of the temporal-order reconstruction task.

Item	Incidental list	category	Item	Intentional list	category
1	table	furniture	1	valley	landscape
2	guitar	instrument	2	shoes	clothes
3	mixer	kitchen utensil	3	pig	animals
4	gun	weapon	4	carrot	vegetable
5	couch	furniture	5	lake	landscape
6	chair	furniture	6	mountain	landscape
7	harp	instrument	7	tie	clothes
8	refrigerator	kitchen utensil	8	cow	animals
9	missile	weapon	9	peas	vegetable
10	pistol	weapon	10	spinach	vegetable
11	desk	furniture	11	canyon	landscape
12	piano	instrument	12	shirt	clothes
13	stove	kitchen utensil	13	rabbit	animals
14	cannon	weapon	14	bean	vegetable
15	trumpet	instrument	15	pants	clothes
16	violin	instrument	16	sweater	clothes
17	oven	kitchen utensil	17	horse	animals
18	lamp	furniture	18	river	landscape
19	tank	weapon	19	corn	vegetable
20	toaster	kitchen utensil	20	cat	animals

*Note:* Underlined words were the three item pairs that belonged to one semantic category. All other items pairs always belonged to two different semantic categories.

Procedure. Before the presentation of the incidental list, no instructions were given. Participants were shown the 20 words one word at a time for six seconds. Words were individually written on DIN A6 green index cards. Ten seconds after the last card, participants were instructed to reproduce the list with the help of a deck of 20 randomly ordered index cards showing one word each.

Before the presentation of the intentional learning list, participants were instructed to remember the order of the presented cards. Again, they were shown 20 index cards. Ten seconds after the last card, participants obtained another randomly ordered card deck in order to reconstruct the noun sequence. In both runs, participants sorted the cards until they were satisfied with their reconstructed order.

Data Analyses. The reconstructed word order for both runs was evaluated assessing *overall performance*, *serial organization*, and *semantic clustering*. Overall performance was operationalized as (a) the Pearson product moment correlation between the participants' individually reproduced order and the actual order of the items. A value of 0 – zero correlation – corresponds to random order reproduction. A value of 1 – perfect correlation – corresponds to perfect order reproduction. Overall performance was also operationalized as (b) the sum of the absolute difference values between each item's remembered position and its actual position in the list. A 0-point difference corresponds to no deviation or perfect performance. A 200-point difference corresponds to maximum deviation or the worst possible performance. Serial organization was defined as the number of item pairs that were remembered in the correct order. Altogether, a maximum of 19 item pairs could have been reproduced correctly for a perfect score.

Finally, semantic clustering was measured as the number of remembered item pairs belonging to the same semantic category. There were two types of semantic clustering: correct and incorrect. As described above, in each list, there were three instances where two nouns of the same semantic category were actually presented as neighboring item pairs. If these item pairs were remembered correctly, these were counted as a correct clustering. For the incidental list, these were couch and chair (items five and six), missile and pistol (items nine and ten), or trumpet and violin (items 15 and 16); for the incidental list, accordingly, these were lake and mountain, peas and spinach, pants and sweater. Further, if participants erroneously clustered two semantically related items that did not occur in the list in neighboring positions, these were counted as clustering errors. For the intentional list, this could have been, for example, valley and canyon (items one and eleven). There were altogether 34 possibilities for incorrect semantic clusterings.

The two types of tasks had a 2 (strategy)×2 (group) factorial design with instructions (incidental, intentional) as a within-subjects factor and group (patients, controls) as a between-subjects factor. The instructions were assumed to trigger different memorization strategies: In the incidental learning condition, individuals were assumed to spontaneously use organizational encoding strategies because items could be semantically grouped together. In the intentional learning condition, individuals could use the same strategy as during the incidental learning condition. However, knowing that a memory test would occur afterwards, they could improve their performance by actively incorporating temporal-order information of the word sequence.

Overall performance, serial organization, and semantic clustering were analyzed using repeated-measures two-way ANOVAs with instructions (incidental, intentional) as a within-subjects factor and group (patients, controls) as a between-subjects factor. We used SPSS (Statistical Package for the Social Sciences, version 14.0.1, SPSS incorporation) to conduct statistical analyses with a significance level of .05 for all two-tailed tests. Post hoc Tukey's *t* tests were used to compare groups individually. Data were tested for normal distribution (Kolgomorov-Smirnov test) and homogeneity of variance (Bartlett's homogeneity test).

#### Script-generation task

**Figure 1:**
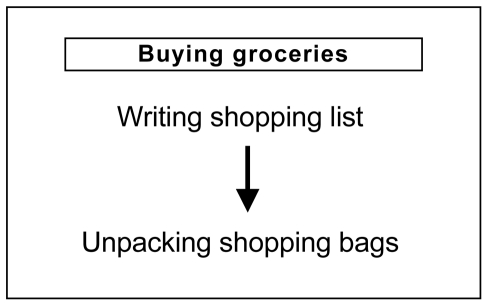
Example of a script card in the script-generation task. *Note.* The heading indicated the theme of the script. The upper event was always a starting item, the lower event was the ending item of the script. The arrow indicates how the participant had to generate the script: downward direction = chronological order (as shown); upward direction = inverse temporal order (not shown). Cards remained visible during script generation. A sample answer of the depicted script “buying groceries” in chronological order could have been: 1) going to the grocery store by car, 2) getting a shopping cart and entering the grocery store, 3) putting groceries into the shopping cart, 4) looking for a register, 5) getting in line, 6) paying at the register, 7) putting the groceries into shopping bags, 8) putting bags into the car, 9) driving home, 10) unloading shopping bags from the car.

Stimuli. For the script task (adapted from, [71]), participants had to generate events of everyday activities (i.e., scripts). Individuals were administered cue cards containing the script header, the starting event, and the final event for two routine scripts and two non-routine scripts [27]. Below the script header (e.g., “buying groceries”), the starting event (e.g., “writing a shopping list”) and the final event (e.g., “unpacking shopping bags”) were printed. Further, there was an arrow between these two events indicating that the participant should generate the script events in chronological (downward arrow) or inverse temporal order (upward orientation) (Figure 1). The experimenter instructed participants to name the events that occur between the starting event and final event according to their temporal order (either in chronological or inverse order).

Procedure. Before the actual testing, participants were trained on two chronological and two inverse script generations. The example scripts were “going out to dinner,” “taking a shower,” “writing a letter,” and “redecorating a room.” After each script generation, participants were shown their generated lists of events and informed about whether they had made mistakes.

Then the testing scripts were administered. These were “doing the laundry” (routine, chronological), “going to a funeral” (non-routine, chronological), “buying groceries” (routine, inverse), and “going to the photographer” (non-routine, inverse). The order of scripts was pseudorandomized across participants. Cue cards remained visible during the corresponding trial. The examiner recorded all events that were generated. There was no time limit.

Data analyses. The script generation task had a 2 (familiarity)×2 (order)×2 (group) factorial design with order (chronological, inverse) and familiarity (non-routine, routine) as a within-subjects factors and group (patients, controls) as a between-subjects factor.

A generation index indicated how much time it took participants to generate events (number of events/total time). Further, three different types of error were assessed: (1) sequencing errors (displacement in the natural sequence of actions within a script: e.g., “drying” before “washing” in the “doing the laundry” script), (2) irrelevant intrusions (actions that did not belong to the script: e.g., “calling the chimney sweeper” in the “doing the laundry” script), and (3) boundary errors (either a failure to begin at the stated starting point of the script or a failure to stop at the stated end point: e.g., “wearing the clothes” in the “doing the laundry” script). For error evaluation, two independent raters (JS and YE) judged the scripts. Inter-rater reliability as assessed by Cronbach's alpha was .96.

The generation index and types of errors were compared for the different order (chronological, inverse) and familiarity (routine, non-routine) conditions between groups (patients, controls) using repeated-measures ANOVAs. Data were tested for normal distribution (Kolgomorov-Smirnov test) and homogeneity of variance (Bartlett's homogeneity test). Subesquent Tukey's *t* tests were used to specify group differences.

### Correlations

Performance measures were correlated (Pearson's correlation coefficient) between tasks, as well as with symptomatology, medication intake, and demographic variables.

## Results

### Temporal-order reconstruction task

#### Overall performance

**Figure 2:**
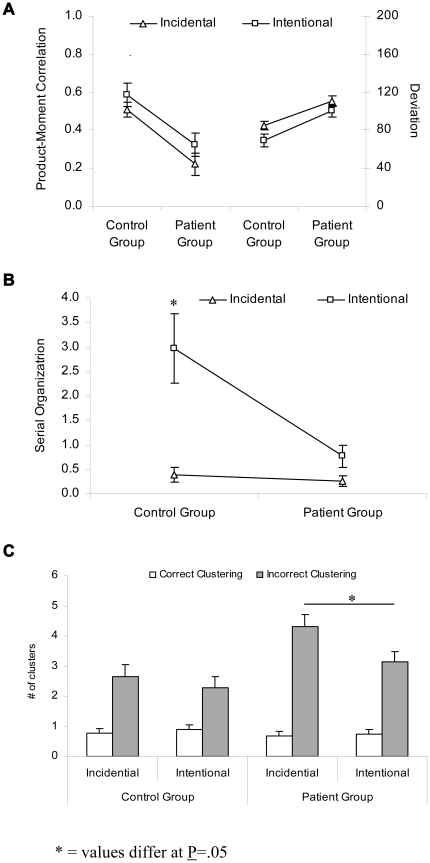
Performance measures of the patient and healthy control group for the temporal-order reconstruction task. a) Overall performance depicted as the Pearson product moment correlation (on the left) and sum of absolute difference deviations (on the right). b) Serial organization depicted as the number of reproduced item pairs. c) Semantic clustering depicted as correct clustering and clustering errors.

Figure 2a shows the overall performance results for the temporal-order reconstruction task. The overall reconstruction performance of both groups was measured as (a) the correlation measure between the remembered and actual order and (b) the deviation measure as the absolute value of the difference between each item's remembered position and its actual position. Regarding the overall performance, the repeated-measures ANOVA for the correlation measure showed a significant main effect of instructions (F(1,66) = 4.72, P<0.033). Overall performance was better in the intentional than in the incidental learning condition for both groups. In addition, there was a significant main effect of group (F(1,66) = 16.98, P<0.001), indicating that controls performed better than patients. The interaction between group and instruction was not significant (F(1,66) = 0.75, P>0.05). Similar results were obtained using the deviation measure for overall performance. There was a main effect of instructions (F(1,66) = 10.15; P<0.002) and group (F(1,66) = 16.75, P<0.001) but no instructions × group interaction effect (F(1,66) = 0.26, P>0.05). This indicates that overall temporal-order reconstruction was worse in patients than in healthy controls for both strategies.

#### Serial organization

Serial organization results are depicted in Figure 2b. It was measured as the number of correctly remembered word pairs (maximum of 19). The repeated-measures ANOVA revealed a significant main effect of instructions (F(1,66) = 19.79; P<0.001) and group (F(1,66) = 7.98, P<0.006) as well as a significant interaction between instructions and group (F(1,66) = 9.05, P<0.004). The post hoc *t* tests showed that controls (t(66) = −2.984, P<0.005) but not patients (t(66) = −0.651, P>0.05) improved in serial organization from the incidental to the intentional learning condition.

#### Semantic clustering

Figure 2c represents the semantic clustering results. Correct clustering means that participants correctly reconstructed two category members in sequential order, whereas clustering errors means that participants incorrectly reconstructed two category members in sequential order that were not presented sequentially at study. The repeated-measures ANOVA on semantic clustering demonstrated a significant main effect of instructions (F(1,66) = 11.98; P<0.01), clustering (F(1,66) = 6.86; P<0.05), and group (F(1,66) = 16.24, P<0.01). Further, there was a significant interaction between group and instructions (F(1,66) = 5.41; P<0.05). The subsequent Tukey's *t* test showed that patients committed more clustering errors than healthy controls in the incidental (t(66) = −2.79; P<0.05) but not in the intentional (t(66) = −1.75; P>0.05) condition. In fact, clustering errors where higher in the incidental compared to the intentional instructions condition only in patients (t(33) = −2.25; P<0.05) but not in healthy controls (t(33) = −1.19; P>0.05).

### Script-generation task

#### Generation Index

Table 3 shows all parameters for the script-generation task. The generation index indicates the relative time per generated event. The repeated-measures ANOVA for the generation index revealed significant main effects of order (F(1,64) = 43.04, P<0.001), familiarity (F(1,64) = 94.49, P<0.001), and group (F(1,64) = 10.52, P<0.01). This implies that healthy controls generated events more quickly than patients across all conditions. Independent of group, all participants showed faster event generation in the routine condition compared to the non-routine condition and in the chronological compared to the inverse condition. The three-way interaction between familiarity × order × group (F(1,64) = 9.47, P<0.01) indicated that the generation of events was fastest in the chronological/routine condition compared to the other three conditions. More importantly, healthy controls had a higher generation index than patients in this condition.

**Table 3:** Performance on the script-generation task separately for patients with schizophrenia and healthy controls.

		Chronological		Inverse	
		Routine	Non-routine	Routine	Non-routine
Generation index	SZ	.18 (.03)*	.11 (.03)	.13 (.04)	.11 (.03)
	C	.26 (.05)*	.14 (.02)	.16 (.02)	.14 (.02)
*Errors*					
Sequencing errors	SZ	1.5 (.20)	3.6 (.27)	4.9 (.56)*	5.1 (.45)*
	C	.52 (.11)	2.0 (.24)	2.2 (.39)*	2.9 (.26)*
Irrelevant intrusions	SZ	.06 (.04)	1.0 (.29)*	.47 (.22)	.17 (.10)
	C	.01 (.01)	.09 (.07)*	.27 (.24)	.21 (.14)
Boundary errors	SZ	.18 (.07)	.32 (.10)	.72 (.14)*	1.1 (.16)*
	C	.06 (.04)	.06 (.03)	.27 (.08)*	.34 (.09)*

Note. N = number of subjects; SZ = chronic schizophrenia patients; C = healthy controls; in brackets = standard errors;

* = groups differ; p = .05.

#### Errors in the script-generation task

Sequencing Errors indicated violations of the chronological temporal order of the script. The repeated-measures ANOVA for sequencing errors revealed a significant main effect of order (F(1,65) = 52.87, P<0.001) and familiarity (F(1,65) = 37.46, P<0.001), as well as a significant interaction between order × familiarity (F(1,65) = 10.62, P<0.002). In both groups, sequencing errors were higher in the non-routine compared to the routine condition for chronological scripts only. The main effect of group (F(1,65) = 37.57, P<0.001) and a group × order interaction (F(1,65) = 5.32, P<0.024) indicated that the number of sequencing errors differed between groups only in the inverse but not in the chronological condition.

Irrelevant intrusions were enumerated events that did not belong to the script. The repeated-measures ANOVA on intrusions revealed a significant order × familiarity interaction (F(1,64) = 5.86, P<0.018). For both groups, intrusions were higher in the non-routine compared to the routine condition for chronological scripts only. There was a main effect of group (F(1,65) = 5.35, P<0.025) and a three-way order × familiarity × group interaction (F(1,65) = 5.86, P<0.018). This indicates that patients made predominantly more intrusion errors than healthy controls when chronological, non-routine scripts had to be generated.

Boundary errors were failures to begin at the appropriate starting point or end at the appropriate ending point of the script. The repeated-measures ANOVA on boundary errors revealed significant main effects of order (F(1,65) = 36.45, P<0.001) and familiarity (F(1,65) = 5.50, P<0.022). Boundary errors were lower in the chronological compared to the inverse condition and in the routine compared to the non-routine condition for both groups. More interestingly, there was a main effect of group (F(1,65) = 9.89, P<0.001) as well as an order × group interaction (F(1,65) = 6.98, P<0.01). This shows that patients made more boundary errors than healthy controls in the inverse conditions only.

### Correlations

For the script task, number of intrusions in the chronological, non-routine condition was significantly correlated with general psychopathology of patients (PANSS general: r ^2^ = 0.54; P<0.01). Further, for patients, age was significantly correlated with number of boundary errors in the inverse, non-routine condition (r ^2^ = 0.50; P<0.01). There were no between-task performance correlations. None of the measures correlated with medication intake dosage.

## Discussion

In this study, we assessed how patients with schizophrenia were able to make use of short- and long-term temporal information facing varying degrees of temporal information availability. We administered a temporal-order reconstruction task and a script-generation task. In the former, patients showed worse overall performance in both the incidental and intentional conditions compared to healthy individuals. In healthy controls, the number of correctly remembered item pairs increased from the incidental to the intentional learning condition. In patients, the number of clustering errors decreased from the incidental to the intentional condition. In the script-generation task, patients compared to healthy controls generated fewer events per time unit in the chronological, routine condition only. Sequencing and boundary errors were higher in patients in the inverse condition for routine and non-routine scripts. Patients generated more irrelevant intrusions than healthy controls in the chronological, non-routine condition only. Overall, these results suggest that patients are able to make use of temporal information of short- and long-term memory. However, the utilization of temporal information from short-term memory leads to a decrease in inappropriate semantic clustering rather than to better memorization of item pairs. Further, utilization of temporal information from long-term memory reveals generation fluency deficits as well as script knowledge imprecision in patients.

Our results confirm that cognitive dysfunctions are a major deficit in patients with schizophrenia [2], [5]–[12], [77], [78]. Nevertheless, former studies have shown that patients' working memory precision [79] and realization of selection [80], [81] are intact, supporting the notion that patients with schizophrenia suffer from impaired access to relevant information.

The present results for the temporal-order reconstruction task support our hypothesis that patients make use of temporal information in a deviant way [16]. In line with functional [39]–[43] and structural anomalies [44]–[47] of the prefrontal cortex in schizophrenia, hypofrontality and prefrontal-hippocampal disturbances might underlie impaired temporal information processing in schizophrenia [17], [82]. For the temporal-order reconstruction task, instructions were used to trigger different memorization strategies. The use of temporal information of the word sequence was assumed to be more pronounced in the intentional than the incidental learning condition. As expected, for the recall of item pairs, healthy individuals were able to make use of this information by increasing the number of remembered item pairs from 0.38 to almost 3 from the incidental to the intentional learning condition. Patients' performance in remembering item pairs, by contrast, did not differ with regard to the two different task instructions. However, patients' clustering error rate improved from the incidental to the intentional condition, resulting in an error rate comparable to that of healthy controls in the incidental condition.

This is in line with the results of Elvevag et al. [13], who found that memory for temporal order and recall of the actual words were highly correlated. The authors argue that a third process might underlie these impairments. As we have argued, patients' cognitive deficits are likely to be a consequence of strategy imprecision [15]. Specifically, patients make use of similar information acquisition and processing strategies independent of task demands. Further, another study showed that brain activity during incidental encoding was more similar between healthy controls and patients with schizophrenia than during intentional encoding [53]. In line with this argumentation, Iddon et al. [51] found that patients' impairments in using adequate strategies comprises not only verbal but also visuospatial mnemonic strategies. This further supports the notion that strategic inflexibility across tasks could help to explain why patients fail on higher order cognitive tasks such as problem solving, planning, or goal-directed behavior [14], [83].

On the temporal-order reconstruction task, the different outcomes due to the incidental and intentional learning strategies represent this cognitive inflexibility. Specifically, the availability of sequential episodic temporal information led to different improvements in patients and controls. Although patients improved their semantic clustering error rate, healthy controls were able to form more memories of item pairs. Hence, temporal information helps patients to overcome inappropriate semantic clustering. This is in line with the desinhibition deficit in schizophrenia and the assumption that task-irrelevant information impedes patients' performances, see, e.g., [84]. Patients have difficulties suppressing an imminent stimulus-driven response (according to semantic content) in favor of a voluntary action (by integrating temporal information). In fact, patients appear to cluster item pairs according to their semantic content rather than according to their temporal sequential occurrence in the first place. Only when temporal information is given explicit (intentional) attention will patients access the temporal information of item sequences. Nevertheless, patients do not or cannot use this information to improve their item memory and retrieval. On the contrary, accessing temporal information helps patients to overcome the biasing influence of semantic content.

Our second task, the script-generation task, assessed the ability to access and process temporal information in long-term memory. The task was specifically designed to differentiate the ability to access (familiarity) and manipulate (temporal order) temporal script knowledge. Surprisingly, patients differed from healthy controls in the speed of generating script events only in the chronological and routine condition. In fact, the speed of retrieval from long-term memory has been found to be impaired in schizophrenia patients [85], [86]. Specifically, the deficit has been found to be larger for semantically compared to phonologically related items, implying that, besides a general long-term memory deficit, patients with schizophrenia might imprecisely encode semantic, in this case temporal, information [87], [88].

This imprecision resulted in a specific error pattern on the script-generation task. Specifically, in the more demanding inverse condition, patients made more sequencing and boundary errors independent of whether the item was familiar or unfamiliar. Sequencing errors are direct time sequence violations that have been associated specifically with disorganization symptoms in patients with schizophrenia [29]. In our inverse script-generation condition, these sequencing errors might have been a consequence of the manipulation of temporal information since the “normal” chronological sequence of events had to be inverted. Further, functional and structural changes of the hippocampus in patients with schizophrenia [48]–[50] could be implicated. Weiss et al. [89] showed that verbal memory impairments in patients with schizophrenia are associated with lower hippocampal activation. Hence, impaired long-term memory recollection in patients, as well as higher task demands in the inverted conditions, are likely to lead to temporal sequence violations.

The increased number of boundary errors also supports this view. Boundary errors indicate a failure to begin at the indicated starting point or to stop at the indicated end point of the script. Former studies have shown that patients with schizophrenia detect boundaries between large action sequences only imprecisely [35]. Interestingly, deficits in organizing and planning action sequences even occur when working memory load is minimized [32], suggesting that in addition to task difficulty, a deficient representation of temporal information in long-term memory might be responsible for the deficient results. Our results fit nicely within this research framework as we show that these boundary errors occur predominantly when temporal information has to be manipulated upon retrieval. As was the case for sequencing errors, the number of boundary errors was not increased in patients in the chronological conditions. In sum, this implies that deficits in the planning of event sequences [63]–[65] or even action monitoring [61] might be accounted for by disturbed processing of temporal information. If task difficulty changes, the deficits in patients become more apparent possibly due to cognitive strategy inflexibility [14], [15]. Consequently, the efficient processing of available temporal information, which has been encoded into and retrieved from long-term memory, might be disturbed in patients with schizophrenia.

Irrelevant intrusions, might, on the other hand, relate more to symptomatology in our patient sample. These errors indicate instances in which events not belonging to the script were, nevertheless, named by the participants. In the chronological and non-routine condition (“going to a funeral”), the number of irrelevant intrusions was higher in patients with schizophrenia compared to healthy controls. In fact, we found a significant correlation between number of intrusions and general psychopathology in this condition in patients, confirming the idea that intrusions may be related to symptomatology. This result may provide evidence for the fact that dissociative thinking in schizophrenia can lead to illusory associations. In fact, reproducing the path of an 800 m closed-loop walk, which participants in the study by Daniel et al. [90] had to walk twice, patients also produced more irrelevant comments, indicating that information not directly related to the spatial route might interfere with correct spatio-temporal information encoding or retrieval. One of the underlying mechanisms of hallucinations of persecution is to misinterpret the intentions of others and to believe in the associative nature of unrelated facts [62], [91]–[96]. Bleuler [97], [98] recognized that dissociating is a basic symptom of schizophrenia, leading to thought incoherence and imprecision in thought expressions. The increased number of intrusion errors of patients in our “going to a funeral” script, therefore, may have been based on the high demands to combine formal and semantic aspects of the script task.

In conclusion, this study provides evidence that patients with schizophrenia process temporal information inefficiently. On the temporal-order reconstruction task, the integration of temporal information from short-term memory in the intentional condition did not improve recollection of item pairs in patients. Instead, it improved patients' semantic clustering. On the script-generation task, patients were slower than healthy controls in generating events only in the chronological routine condition. In addition, increased sequencing and boundary errors in the inverse condition indicate that temporal information retrieved from long-term memory was processed ineffectively by patients. Finally, the higher rate of intrusion errors in the chronological and non-routine condition can be associated with formal thought disorder and general psychopathology in patients. Future work should investigate the underlying neural correlates of the revealed deficits in patients with schizophrenia. From a clinical point of view, these results suggest that temporal information can be used by patients but only in an imprecise and strategically inefficient way. This provides insight into the everyday difficulties encountered by these individuals.
